# AI Painting Effect Evaluation of Artistic Improvement with Cross-Entropy and Attention

**DOI:** 10.3390/e27040348

**Published:** 2025-03-27

**Authors:** Yihuan Tian, Shiwen Lai, Zuling Cheng, Tao Yu

**Affiliations:** 1 Culture Design Lab, Graduate School of Techno Design, Kookmin University, Seoul 02707, Republic of Korea; tianyihuan@kookmin.ac.kr; 2 Department of Global Convergence, Kangwon National University, Chuncheon-si 24341, Republic of Korea; lswknu@kangwon.ac.kr (S.L.); 9710136280046@kangwon.ac.kr (Z.C.); 3 Department of Smart Experience Design, Kookmin University, Seoul 02707, Republic of Korea

**Keywords:** MCASVM, art creation, cross-entropy, probabilistic calibration network

## Abstract

With the rapid development of AI technology, AI painting tools are increasingly used in art creation. However, the effects of works created by different users using AI painting tools vary. Finding out the factors that affect the level of art creation after users use AI painting tools is a matter of concern. To solve this problem, this paper proposes a new Multi-Classification Attention Support Vector Machine (MCASVM) with cross-entropy loss function. By identifying and predicting the level of creativity of ordinary users after using AI painting tools, this model compares and analyzes the influencing factors behind the high and low effects of artistic creativity enhancement after using AI painting tools. The main contribution of this paper is to establish the Art Creation Ability Assessment Dataset (ACAAD) through real data collection to provide data support for subsequent assessments. Meanwhile, MCASVM directly handles the multi-classification problem in the established dataset by introducing multiple SVMs. Among other things, the probabilistic calibration network adjusts the model output so that its predicted probabilities are closer to the probability that the sample truly belongs to the classification. DBAM enhances the feature fusion capability of the model by explicitly focusing on the important channel and spatial features, and it enables the model to more accurately recognize and differentiate between changes in the creative abilities of different users before and after using AI painting tools. The experimental results show that the artistic creativity of ordinary users can be enhanced by AI painting tools, where the most central influencing factors are interest level and social support.

## 1. Introduction

With the rapid advancement of AI technology, particularly breakthroughs in computer vision, entropy, and information theory [1,2], the application of AI in artistic creation has become an important topic of academic and public interest. In recent years, the widespread adoption of AI painting tools, especially among ordinary users, has sparked extensive academic discussions and practical explorations regarding their potential to enhance artistic creativity. These tools not only provide a fast and convenient way to create art but also help users overcome creative bottlenecks and inspire inspiration. AI painting tools such as DALL-E, Stable Diffusion, and MidJourney utilize deep learning algorithms to generate high-quality images. These tools can analyze and understand a large number of artworks, learn styles and techniques, and subsequently incorporate these elements into new creations. This ability makes AI painting tools powerful assistants for artists exploring new styles and techniques. Additionally, AI painting tools are highly customizable, allowing users to adjust creative parameters according to their needs and, thus, create unique works of art. This convenience has attracted significant attention from creators, providing artists with new avenues for inspiration and creative exploration. These features collectively promote the integration of AI and artistic creation, opening up new possibilities for the development of art.

Nevertheless, despite the growing body of research in this area, questions remain about how to systematically evaluate the impact of AI painting tools on users’ artistic creativity and the underlying mechanisms through which these impacts occur [3]. The enhancement of artistic creativity is influenced by a complex interplay of factors, including individual differences, the context of usage, and specific creative objectives [4]. The traditional evaluation of artistic creation ability mostly relies on expert reviews or subjective evaluations and lacks systematic quantitative analysis. Therefore, the goal of this paper is to establish a more objective and accurate deep learning evaluation model to evaluate the improvement effect of ordinary users’ artistic creation abilities after using AI painting tools [5]. Specifically, this paper intends to solve the following two key problems.

Question 1 is, how do we assess the impact of AI drawing tools on improving the artistic creativity of ordinary users? We used a quantitative assessment to determine the changes in ordinary users’ ability to create art before and after using the AI drawing tool. Then, we analyzed the responses of different user groups to the AI drawing tool [6]. Question 2 is, what factors influence the level of users’ art creation ability? First, the factors that may affect the effect of art creation, such as users’ basic characteristics (e.g., age, gender, educational background, etc.), motivation to create, creative environment, and social support, were discussed. And based on this, a dataset was established. Finally, through example validation, we explored how these variables affected users’ creative effects after using AI drawing tools [7].

To solve the above two problems, this paper created the Art Creation Ability Assessment Dataset (ACAAD) based on real data and proposed the Multi-Classification Attention Support Vector Machine (MCASVM). MCASVM creatively applies machine learning methods, especially SVM and attention mechanism, to the assessment of artistic creation ability. In the traditional assessment of artistic creativity ability, the factors affecting artistic creativity ability are often not paid attention to [8]. By introducing machine learning algorithms, this study provides more objective and accurate evaluation results, which help to understand the specific impact of AI painting tools on the improvement of artistic creation ability [9]. At the same time, this paper combines multidimensional influencing factors for analysis. These factors include not only the personal characteristics of users but also external factors such as creative environment and social support, reflecting a multidimensional understanding of artistic creation ability [10]. This research perspective helps to deeply explore the differences in the creative performance of different types of users when using AI painting tools, thereby providing a theoretical basis for the optimization and personalized customization of AI painting tools. The main contributions of this paper are mainly reflected in the following three aspects:

(1) By collecting information from real-life AI painting users, this paper creates a multidimensional Art Creation Ability Assessment Dataset (ACAAD), which provides a basis for the subsequent identification and prediction of factors behind the enhancement of artistic creativity of ordinary users;
(2) A Multi-Classification Attention Support Vector Machine (MCASVM) with a cross-entropy loss function is proposed. It innovatively introduces multiple SVMs to deal with multiclassification problems directly and combines the attention mechanism of deep learning with the traditional SVM, utilizing the neural network structure to realize the “one vs. rest” strategy of SVM;
(3) The novel dual-branch attention module (DBAM) aims to fully integrate features of different dimensions. It consists of two branches: the Channel Attention Module and the Spatial Attention Module. DBAM is able to capture the important features in the input data more comprehensively and, thus, improve the model’s classification performance on the multi-factors affecting artistic creativity.

## 2. Related Work

Artistic creativity involves the influence of a variety of factors, and at present, there are relatively few studies on these factors to assess the works they create, and they are assessed in traditional ways such as field research and questionnaires. In this paper, after establishing a dataset based on questionnaire research, we innovatively utilize both support vector machines and attention mechanisms to assess the effect of improving artistic creativity. The following is based on related research work in three main areas: support vector machines, attention mechanisms, and factors affecting artistic creativity.

### 2.1. Support Vector Machine

In recent years, support vector machines (SVMs), an effective machine learning technology, have been extensively studied and applied in many different domains. A variety of optimization models in SVMs and their applications in the economic field have pushed forward the development of the theory of optimization-based SVMs and the research on economic applications [11]. Sebald and Bucklew [12] verified through simulation studies that the performance of SVM is comparable to that of neural networks in dealing with nonlinear problems and further introduced decision feedback processing to cope with the problem of temporal correlation of input vectors generated by inter-symbol interference (ISI) data, which provided a new idea for nonlinear equalization. A method that combines SVM with deep learning utilizes SVM’s interval-based loss function by using a linear SVM instead of a softmax layer in the top layer of a deep network [13]. Based on the experimental results, this approach significantly improves performance across a variety of datasets, offering a fresh approach to deep learning model optimization. Wavelet Support Vector Machine (WSVM) [14] combines wavelet technology with SVM by constructing a wavelet kernel, and the experimental results demonstrate the feasibility and effectiveness of WSVM in regression and pattern recognition, expanding the application scope of SVM. An SVM-based decision tree method generalizes the key ideas of statistical learning theory and SVM to decision trees, and its ability to generate logically simple decision trees with good generalization ability provides a new perspective on the development of decision tree algorithms [15]. Reduced Support Vector Machine (RSVM) [16] explored its performance and utility in dealing with large-scale datasets, and it was found that RSVM has an advantage in training time but is slightly lower than standard SVM in testing accuracy. An SVM training method based on successive over-relaxation (SOR) [17], which is capable of handling large-scale datasets with millions of data points and the algorithm converges faster than other methods, provides an effective tool for the solution of large-scale data classification problems. An enhanced SVM training algorithm [18] offers technical support for the use of SVM on large-scale datasets, can handle a large number of support vectors in large-scale datasets, and is independent of the number of support vectors expected. The development of SVM applications in big data is further advanced by Suthaharan [19], who elaborates on the application of SVM to large-scale data classification problems by streamlining the theoretical and implementation process so that readers may better comprehend and apply SVM. Together, these studies promote the development of SVM theory and the expansion of applications, providing rich ideas and methods for solving complex machine learning problems.

### 2.2. Attention Mechanisms

In the realm of deep learning, attention mechanisms have gained widespread recognition and use, and they are now one of the most important methods for enhancing model performance [20]. A unifying framework for attention models was established by Niu et al. [21]. Additionally, it has conducted comparable research with Flavius Frasincar and Gianni Brauwers [22] to classify current attention models according to standards, including output representation, input feature form, input softness of attention, and input representation. An attention mechanism [23] specialized for fine-grained image recognition tasks learns to pay attention to critical regions in an image by introducing an attention module in the convolutional feature activation layer without additional labeling information. It effectively improves the classification accuracy and achieves excellent performance on several benchmark datasets. A global attention mechanism [24] enhances cross-dimensional interactions by preserving channel and spatial information. According to experimental results, this approach performs noticeably better on image classification tasks than several other attention mechanisms currently in use, indicating that global attention mechanisms have the potential to improve deep neural network performance. For the remote sensing picture classification problem, Cai and Wei [25] suggested an approach based on graph convolution and a cross-attention mechanism. By incorporating the attention mechanism and graph convolution into the feature extraction process to fully utilize the relationship between features, this technique increases the classification accuracy of remote sensing images. By learning spatial attention scores on shallow and deep features of both style and content images and normalizing content features appropriately, an adaptive attention normalization module [26] accomplishes fine-grained style feature migration in the context of arbitrary style migration. To further enhance the stability and quality of style migration, a novel local feature loss function is also suggested. Li et al. [27] proposed a convolutional neural network with an attention mechanism to address the occlusion problem in facial expression recognition. By introducing a gating unit to automatically perceive the occluded regions of the face and focusing the attention on the non-occluded and discriminative regions, the network significantly improves the recognition accuracy of partially occluded facial expression images. A DA-CapsNet [28] enhances the contribution of important information in the capsule and improves the capsule hierarchy by adding an attention module after the convolutional and main capsule layers, respectively, which makes DA-CapsNet outperform the traditional CapsNet on multiple image classification tasks. By comparing the effects of various attention factors and computational techniques on performance in a range of applications, Zhu et al. [29] empirically studied spatial attention mechanisms in deep networks and discovered some results that defy conventional wisdom. For example, the Transformer attention module’s comparison of query and key content is insignificant for self-attention. Attention Flows [30] is an approach for a deeper understanding of the attention mechanisms in Transformer-based language models. This method supports users with visualization tools to query, track, and compare the flow of attention between different layers and attention heads in the model. The Pyramid Attention Pooling Module (PAP) and the Pooled Index Correction Module (PIC), along with an enhanced ResNet-101 backbone network and several data enhancement techniques, are introduced in a semantic segmentation network (SSAtNet) [31] based on the attention mechanism to improve the performance of semantic segmentation of high-resolution remote sensing images.

### 2.3. Factors Affecting Artistic Creativity

Artistic creativity is a complex process that is influenced by a variety of factors, which are generally integrated. In general, many factors are integrated and work together in the whole process of art creation, and artists need to consider them comprehensively in the process of art creation to achieve creative breakthroughs. Niu and Sternberg’s research [32] points out that different cultural backgrounds will have different impacts on artistic creativity. For example, Western culture emphasizes individualism and self-expression, which makes Western artists more inclined to show their unique perspectives and emotional experiences. While Eastern cultures emphasize collectivism and harmony, Eastern artists pay more attention to the integration of their works with society and nature, as well as the cultural connotations and moral meanings carried by their works. The artist’s personality traits are also an important intrinsic factor that influences artistic creation [33]. Some openness to experience will prompt artists to be curious about novelty and willing to experiment with different artistic styles and methods of expression, thus promoting artistic innovation. Meanwhile, intelligence and cognitive ability provide artists with a rich knowledge base and logical thinking ability, enabling them to better conceptualize and plan the structure and content of their works during the creative process [34]. In addition, extroversion also has an impact on artistic creativity. Extroverted artists are better at communicating and collaborating with others and can obtain inspiration and feedback from multiple sources, which facilitates the development of artistic creativity. The social environment can provide external conditions and stimulation for artistic creation. A social atmosphere that values art and encourages innovation can provide a more relaxed and free space for artistic creation [35]. In addition, the motives and emotions generated by the artists themselves are also important psychological factors driving artistic creation, and positive emotions and strong creative motives can promote the creative expression of artists [34]. Education and learning experiences, on the other hand, provide artists with the cultivation of intellectual skills and aesthetic thinking, and interdisciplinary learning and multicultural exposure further expand creative horizons [36]. Mary-Anne Mace et al. [37] constructed a model describing the process of art creation through field observations and interviews to understand the actual behaviors of artists in the art creation process. Teresa M. Amabile [38] experimented to investigate how creativity is affected by outside evaluation. The results of which indicated that participants indicated higher artistic creativity when given specific guidance on artistic creativity and that participants were likely to be less creative and intrinsically interested when externally evaluated. Hu et al. [39] assessed the effects of different assessment styles (anticipatory and feedback assessments) and creative self-efficacy (CSE) as independent variables in a study of the effects of different assessment splits on the artistic creativity of subjects with different CSEs. The results of this study showed that CSE had a significant moderating effect on the impact of assessment on artistic creativity, especially on the dimensions of likability, creativity, and originality. Xue et al. [40] explored the effects of extrinsic motivation on secondary school students’ creativity in science and art. It was found that expected material rewards had a significant negative effect on the scientific creativity of seventh-grade students, while expected social rewards had a significant positive effect on the scientific creativity of eighth-grade students. For artistic creativity, both expected material rewards and expected social rewards had significant positive effects on all students. In addition, teacher and peer evaluations had a significant negative effect on scientific creativity but a significant positive effect on artistic creativity. Some studies focused on the factors that influence children’s artistic creativity. In a study of 60 children’s development of artistic talent and creativity in a drawing program, age was found to be significantly related to knowledge in drawing tasks, but the relationship with problem identification, idea generation, and evaluation varied from task to task [41]. After controlling for technical proficiency, age showed a curvilinear relationship with drawing novelty. Expression and composition played an important role in predicting novelty. Problem-finding ability varied across drawing contexts and interacted significantly with age and evaluative ability. This suggests that the development of artistic creativity involves multiple factors, including motivation, knowledge, problem-finding, evaluation, and age. In addition, it has been shown that children’s creativity shows a U-shaped curve, with a decline in middle grades [42]. Extrinsic factors such as test-based education, extrinsic motivation, and parental emphasis on obedience inhibit creativity, while intrinsic factors such as low cognitive control enhance creativity.

## 3. Method

**Figure 1:**
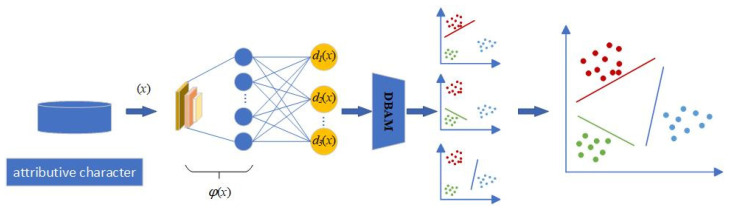
The structure diagram of Multi-Classification Attention Support Vector Machine (MCASVM).

To address the enhancement effect of AI painting tools on the artistic creation ability of ordinary users and the corresponding influencing factors, we propose a Multi-Classification Attention Support Vector Machine (MCASVM). Its overall structure is shown in Figure 1. The initial input of this model is the Art Creation Ability Assessment Dataset, the attributive character of the core information book. These data are then passed through the convolutional neural network to output the feature vector ϕ(x). Next, the DBAM module performs feature fusion after transforming the feature vector’s dimensionality ϕ(x). Finally, the classification results are obtained by a Probabilistic Calibration Network.

### 3.1. Overall Structure of MCASVM

Multi-Classification Attention Support Vector Machine is a deep learning framework model based on SVM [43], which can directly deal with multi-classification problems. As shown in Figure 1, feature extraction is carried out through hidden layers. Multiple SVMSs are used to replace the output layer of the network to complete multiple classification tasks, and each neuron is used to solve a hyperplane. The task of each hyperplane is to divide a class of samples from the sample space. In the output layer of the model, the functional distance of the output of the kth neuron is defined as Equation (1).

(1) $$d_{k}\left(x;\theta_{k}\right)=w_{k}^{T}\varphi \left(x\right)+b_{k}$$

where the output $d_{k}$ of the model represents the functional distance between the sample and each hyperplane, and the parameters of the k hyperplane are represented by $w_{k}^{T}$ and $b_{k}$, and $\theta_{k}(\emptyset ,w_{k},b_{k})$ is used to represent all parameters of the model to be solved uniformly. The task of each of these neurons is to find a hyperplane [44] that can be used to separate one class of samples from the others. To accomplish the above objectives, the loss function is defined as Equation (2).

(2) $$L_{MCASVM}=\sum_{k=1}^{K}\left(\frac{1}{2}{\left\|w_{k}\right\|}^{2}+C\sum_{i=1}^{N}max\left(1-I_{k}\left(y_{i}\right)*d_{k}\left(x_{i}\right),0\right)\right)$$

Among them,

(3) $$I_{k}\left(y_{i}\right)=\left\{\begin{array}{c} 1,y_{i}=k\\ -1,y_{i}\neq k \end{array}\right.$$

Similar to the loss function of SVM, MCASVM is equivalent to adding the generation of multiple hyperplanes based on SVM and finally achieves the purpose of multi-classification by calculating the summation term of the loss of a class of samples divided by each hyperplane. The model combines the “one vs. rest” strategy [45] and the idea of a support vector machine to find the maximum interval hyperplane. It is then implemented through the structure of a neural network, which fully utilizes the advantages of these methods.

If a new sample is given, the category of the new sample is determined by the distance from the sample to the hyperplane. The specific process is shown in Equation (4).

(4) $$\hat{y}={max}_{k}\left(d_{1}\left(x_{i}\right){,d}_{2}\left(x_{i}\right),\ldots {,d}_{k}\left(x_{i}\right)\right)$$

The decision method is similar to the cross-entropy loss function classification [46], which takes the maximum value of network output as the category of sample prediction. Since the task of each hyperplane in the model is to separate one class of samples from the other classes of samples, the test samples should be in the positive class of one class of hyperplanes with a positive functional distance from that class of hyperplanes. And in the negative class of other classes of hyperplanes, the distance from the function of other classes of hyperplanes is negative. Therefore, the method is very simple to determine the category of a new sample point. It is only necessary to look at the functional distance between the sample point and each hyperplane and select the largest value to find the category of the sample.

However, unlike classification using the cross-entropy loss function, this model outputs a functional distance rather than a probability, and it is not possible to intuitively compare the likelihood of samples belonging to different classes from the model output. Considering further optimization of the model output, a Probabilistic Calibration Network is proposed to improve this problem.

### 3.2. Probabilistic Calibration Network

This section proposes probabilistic calibration networks to handle the output of the classification model. Probability calibration is proposed to optimize the model output so that the sample’s probability predicted by the model is as close as possible to the probability that the sample belongs to this class. We first introduce the goal of probabilistic calibration, i.e., the perfect calibration. Suppose the output of the network is $\hat{p}\left(x\right)=\left({\hat{p}}_{1}\left(x\right)\right),\ldots ,{\hat{p}}_{k}\left(x\right),\forall \hat{p}\in [0,1]$, we take $\hat{p}=max\left(\hat{p}\left(x\right)\right)$ as a model to forecast probability sample, the corresponding forecast category is $\hat{Y}={\hat{y}}_{i}$. The following formula defines the perfect calibration as shown in Equation (5).

(5) $$P\left(\hat{Y}=y|\hat{P}=p\left(x\right)\right)=p\left(x\right)$$

where y represents the true class of the sample, and p represents the true probability that the sample belongs to that class. For example, if the model predicts that each of the 10 samples has an 80% probability of being in the positive class, then there should be 8 samples that can be classified correctly. If the output of the model to be calibrated is not a probability, it is converted to a probability output by Equation (6).

(6) $${\hat{p}}_{i}=\sigma {\left(s_{i}\right)}^{\left(k\right)}=\frac{exp\left(s_{i}^{\left(k\right)}\right)}{\sum_{j=1}^{K}exp\;s_{i}^{\left(j\right)}}$$

where $s_{i}$ represents the prediction score of the model output, and $\sigma \left(\cdot \right)$ represents the processing of the model output. In short, probabilistic calibration is obtained by using the output and label of the model $s_{i}{,\;\hat{p}}_{i},\;y_{i}$, and calculating the probabilistic calibrated output ${\hat{q}}_{i}$. Calculate the calibrated output ${\hat{q}}_{i}$.

Platt [47] scales by looking for the scalars a,b to scale the output, as shown in Equation (7).

(7) $${\hat{p}}_{i}=\sigma {\left(as_{i}+b\right)}^{\left(k\right)}$$

Temperature calibration [48] is achieved by looking for the scalar parameter T to scale the output, as shown in Equation (8).

(8) $${\hat{p}}_{i}=\sigma {\left(s_{i}/T\right)}^{\left(k\right)}$$

After the calibrated probability output is obtained, the calibrated probability is obtained by the maximum value of the output after scaling, as shown in Equation (9).

(9) $$q_{i}=max{\hat{p}}_{i}$$

It can be seen that the probabilistic calibration method does not change the predicted results of the model and outputs with large values are scaled by probabilistic calibration. In addition, probabilistic calibration can reduce the instances where the original model outputs are over-confident or under-confident by scaling the output in this way.

The implementation of the probabilistic calibration network is accomplished by using a single 1 × 1 convolution kernel in the convolutional layer, the structure of which is shown in the figure below. If the convolution kernel is temperature-calibrated, only one variable is set. If the convolution kernel is calibrated by Platt scaling, another offset item needs to be added to the convolution kernel. The convolution kernel is used to scale the model output by moving the convolution kernel, which is the concept of shared weights in a convolutional neural network.

**Figure 2:**
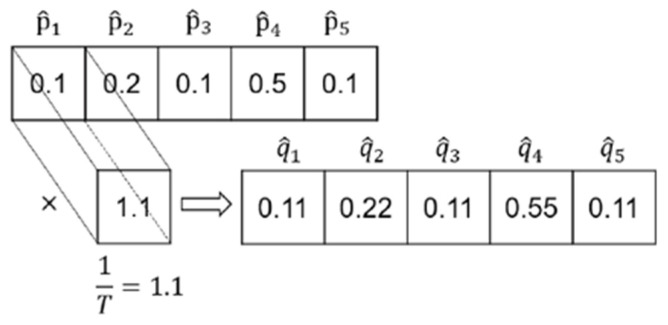
Implementation of probabilistic calibration.

As shown in Figure 2, before calibrating the output of the model, the model prediction sample belongs to the fourth category. After a convolution kernel calibration, the sample is also judged to belong to the fourth class. The probabilistic calibration network does not change the prediction results of the model but enhances the reliability of the model’s own judgment.

All in all, the probabilistic model is completed by only one convolutional layer, which is used to complete the parameter calculation of the probabilistic calibration model. That is, the probabilistic calibration network essentially extends the probabilistic calibration methods of Platt scaling and temperature scaling into the deep learning framework. Unlike the original calibration model, the parameters are computed through the network to avoid memory overflow errors when dealing with large sample data.

### 3.3. Double-Branch Attention Module

**Figure 3:**
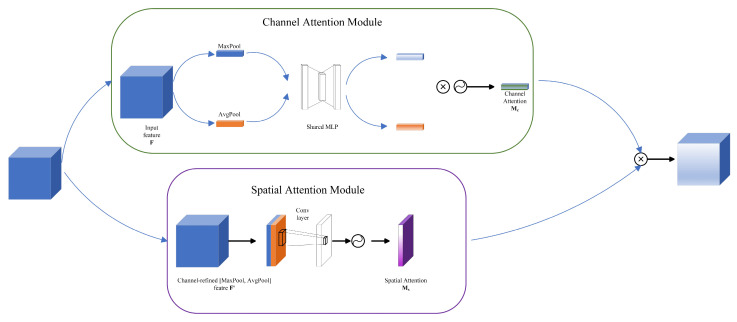
DBAM structure diagram.

After the features were obtained by the multi-layer perceptron, this paper designed a Double-Branch Attention Module to enhance the feature fusion capability of the model and improve the classification performance of the model. Its specific structure is shown in Figure 3. DBAM consists of two branches: the Channel Attention Module and the Spatial Attention Module.

In the above branch channel, the input feature maps of the attention module are, firstly, MaxPool and AvgPool operations and two different groups of feature maps are obtained, respectively. Then, the two sets of feature maps are passed into a Shared multi-layer perceptron (Shared MLP) for feature fusion, and the two feature maps are output. Next, the two feature graphs are multiplied by an element-by-element operation to produce a Channel Attention $M_{c}$.

The branch space attention module below receives the Channel-refined feature $F^{\prime }$ (channel-refined feature $F^{\prime }$) from the channel-refined Feature module, which is further refined through MaxPool and AvgPool. These processed feature maps are then used for spatial feature extraction through a Conv layer. Finally, the feature maps output by the convolutional layer are generated by an activation function to generate Spatial Attention $M_{s}$. The channel attention feature map ($M_{c}$) is combined with the spatial attention feature map ($M_{s}$), and the final feature map is obtained by element multiplication. In essence, DBAM improves the performance and accuracy of the overall model by explicitly focusing on important channel and spatial features.

In this paper, the loss function was selected as the cross-entropy loss function combined to optimize the parameters of the model, which is shown in Equation (10).

(10) $$L=-\frac{1}{N}\;\sum_{i=1}^{N}\sum_{c=1}^{k}y_{ic}\;logp_{ic}\;$$

where N is the number of samples and k is the number of categories. $y_{ic}$ equals 1 when the category of the *i*th sample is c; otherwise, $y_{ic}$ equals 0. $p_{ic}$ is the predicted probability that the category of the *i*th sample is c.

### 3.4. Algorithm Implementation

The MCASVM implementation in Algorithm 1 completes the generation of hyperplanes by modifying the loss function. First, our model extracts features through a multi-layer perceptron (MLP) and then processes the features using maximum pooling and average pooling operations. After the processing, a shared MLP and Sigmoid function are used to generate an attention mechanism for adjusting the features. Immediately after that, the model performs another pooling operation on the tuned features and generates the final attention weights using convolution and Sigmoid functions. Finally, the features are weighted using these weights and classified by the Softmax function to output the final prediction. The whole process also includes the control of counters to ensure that the appropriate operations are performed at different steps.
**Algorithm 1:** Pseudocode of DBAM-MCASVM-PCN Model.**Input:** Input features
$x_{1},\;x_{2},\;\ldots ,\;x_{n}$
**Output:** Classified result
y
Residual =
∅; counter =
∅
layers = [MLP Feature Extraction, DBAM Module, MCASVM Classifier, Probabilistic Calibration Network]1: **While** counter < len(layers) ∗ 2 + 1:2:      **If** counter < len(layers):3:         **If** counter == ∅:4:          layer = MLP Feature Extraction5:         **ElseIf** counter == 1:6:          
$F_{max}=Maxpool(F)$
7:          
$F_{avg}=Avgpool(F)$
8:          
${MLP}_{F_{max}}={Share}_{MLP(F_{max})}$
9:          
${MLP}_{F_{avg}}={Share}_{MLP(F_{avg})}$
10:          
$M_{c}=Sigmoid({MLP}_{F_{max}}*{MLP}_{F_{avg}})$
11:          
$F^{\prime }=M_{c}*F$
12:          
$F_{max}^{\prime }=MaxPool{(F}^{\prime })$
13:          
$F_{avg}^{\prime }=AvgPool{(F}^{\prime })$
14:          
$M_{s}=Sigmoid(Conv(F_{max}^{\prime }+F_{avg}^{\prime }))$
15:          
$F_{final}{=M}_{s}*M_{c}*F$
16:       **ElseIf** counter == 2:17:          d = []18:          For each class k in {1, 2, …, K}:19:              $d_{k}=$ $w_{k}$ ∗ $F_{final}$$+b_{k}$
20:              Append $d_{k}$ to d
21:       **ElseIf** counter == 3:22:          *p = Softmax(d)*23:          *y = argmax(p)*24:           **EndIf**25:           *x* = layer*(x)*26:       **Else:**27:            **If** counter == len(layers):28:              layer = MCASVM Classifier29:            **ElseIf** counter == len(layers) + 1:30:              layer = Probabilistic Calibration Network31:            **EndIf**32:            *x* = layer*(x)*33:          counter = counter + 134: *y* = Output Layer*(x)*
35: **Return** y

Through the backpropagation algorithm of the neural network, the multi-classification task on one network is completed directly. The difference between this method and the cross-entropy loss classification is discussed in detail here. When the loss function $L_{MCASVM}$ calculates the gradient, the contribution of different samples to the parameter update is different. For samples $I_{k}\left(y_{i}\right)*d_{k}\left(x_{i}\right)\geq 1$, that is, samples that do not need to be punished in the SVM method idea, the gradient is shown in Equation (11).

(11) $$\left\{\begin{array}{c} \frac{\partial L}{\partial w_{k}}\sum_{k=1}^{K}w_{k}\\ \frac{\partial L}{\partial b_{k}}=0 \end{array}\right.$$

For those samples, $I_{k}\left(y_{i}\right)*d_{k}\left(x_{i}\right)<1$, the samples in the penalty term should be calculated in the original SVM method, and the gradient is shown in Equation (12).

(12) $$\left\{\begin{array}{c} \frac{\partial L}{\partial w_{k}}\sum_{k=1}^{K}\left(w_{k}-\sum_{i=1}^{N}CI_{k}\left(y_{i}\right)\varphi \left(x_{i}\right)\right)\\ \frac{\partial L}{\partial b_{k}}=-\sum_{k=1}^{K}\sum_{i=1}^{N}CI_{k}\left(y_{i}\right) \end{array}\right.$$

By comparing Equation (5) and Equation (6), when calculating the gradient, samples in different positions have different influences on the model loss function. The model should focus not only on the sample points that need to be trained but also on those samples that are more likely to affect the decision hyperplane. This property enhances the prediction ability and generalization performance of the model.

## 4. Experiment and Analysis

### 4.1. Dataset Introduction

To investigate the impact of AI painting tools on the artistic creation abilities of ordinary users and the corresponding influencing factors, this paper presents the Art Creation Ability Assessment Dataset (ACAAD), which was compiled by sampling from various demographic groups. It primarily describes the characteristics and effects associated with different AI painting users. The main structural information is summarized in Table 1, where the training set comprises 10,000 users, and the test set includes 2000 users. According to the factors affecting artistic creativity mentioned in Section 2.3, it can be known that it includes the following main factors: the Chinese and Western cultures to which the artist is subjected, personality, creative motivation, emotional positivity, social environment, intellectual and cognitive ability, educational experience and background, and external evaluation. And our dataset is divided into six dimensions. Among them, degree generally corresponds to educational background and intellectual and cognitive abilities; artistic motivation corresponds to the motivation to create art; interest level represents the creator’s emotional motivation to create art, and social support corresponds to the social environment as well as external evaluation.

By selecting different features and coding independent variables, we assessed the impact of the AI drawing tool on the users’ artistic abilities and the factors that influenced them. First, we collected the paintings created by these 12,000 users three months before and after using the AI painting tool. Further, we asked a professional painting teacher to rate the paintings they created based on their color scheme, line usage, compositional layout, and expression of mood, with a total score of 100. When the score of the paintings created before and after three months increased by more than 15 points, they were categorized as having a significant impact. If the improvement in score is between 0 and 15 points, it is classified as a moderate impact. If the score does not increase but decreases, it is classified as a negative impact.

In order to validate the generalization performance of the model and to compare it with other state-of-the-art models. In this paper, experiments are also conducted on another publicly available art assessment dataset. Boldbrush Artistic Image Dataset (BAID) [49] is a large dataset for aesthetic assessment of art images. The dataset contains 60,337 artworks encompassing a variety of art forms, such as paintings and drawings, and has collected over 360,000 votes from online users. These votes reflect people’s aesthetic preferences for artworks, with a higher number of votes indicating that the work is considered more aesthetically pleasing. The construction of the dataset is based on a monthly art contest on the Boldbrush website, in which participating artists upload their works, and users can vote for their favorite works, and this voting mechanism makes the ratings in the dataset highly credible and authoritative.

### 4.2. Comparison of Experimental Results

The ACAAD proposed in this paper is characterized by a small data volume and dimensionality. In its classification approach, the depth model employs a fully connected neural network structure. By comparing the hidden layer designs of a one-layer fully connected network and a two-layer fully connected network, we explore the classification accuracy of the traditional Deep Neural Networks (DNN) [50] method and the MCASVM. The specific experimental results are presented in Table 2 and Table 3. As shown in Table 2 and Table 3, the MCASVM achieved superior results on the ACAAD.

After that, we used MCASVM to classify ACAAD, a multidimensional dataset, based on these three results. The classification accuracy of the model was first verified. Then, from the classification results, we observe and count the percentage of users of different dimensions in the three sections: reduce, no difference, and rise. It is known that there are three results in artistic creation ability after using AI painting tools: significant improvement, moderate improvement, and negative improvement. We will record the results with significant improvement effects in Table 4 as “rise”, the results with mild improvement effects as “no difference”, and the results with negative improvement effects as “reduce”. From there, we can determine which factors will enhance the art creation ability after using AI painting tools.

The experimental result in Table 4 indicates the proportions of consistent, decreased, and increased effects on artistic creation ability improvement before and after using the tools. Notably, the two factors of interest level and community support significantly enhance the improvement in artistic creation ability. Therefore, we conclude that AI painting tools can substantially enhance the artistic creation abilities of ordinary users, with interest level and community support identified as the core influencing factors.

To better validate the generalization performance of MCASVM, we verified the accuracy of the model’s classification recognition on another art evaluation dataset, BAID. We also compared the experimental results with two other state-of-the-art models, SAAN [49] and TANet [51], on this dataset. As shown in Table 5, SAAN has the highest classification accuracy of 0.7680 on the BAID dataset. DNN, as well as traditional SVM, have poor classification accuracy. The classification accuracy of our proposed model, MCASVM, although a little lower than SAAN, still exceeds TANet and the other two models. This is sufficient to show the effectiveness and generalization performance of our proposed model.

### 4.3. Visualization of the Model

In the visualization component of the model, the sklearn library [52] was used to generate spiral data for comparison, thus demonstrating the classification effect of more complex datasets.

**Figure 4:**
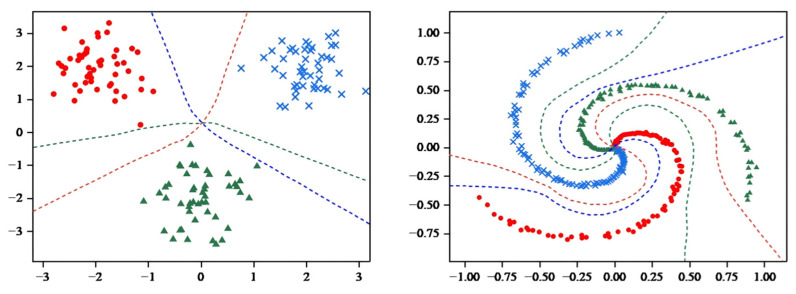
MCASVM generates hyperplane diagram.

As shown in Figure 4, MCASVM generates three hyperplanes based on three classes of datasets, where each class of hyperplanes separates one class of sample points from the others in the sample space. To illustrate the classification effects more clearly, the decision hyperplane and classification regions were plotted using the decision function Equation (4).

**Figure 5:**
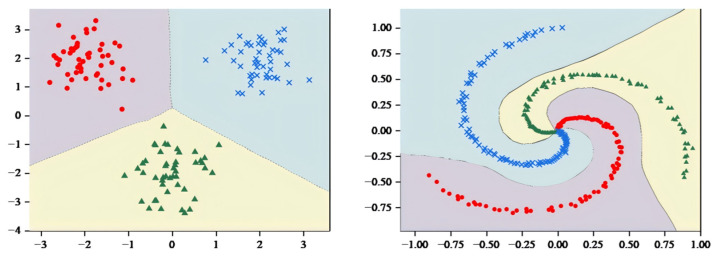
MCASVM divides the sample space diagram.

As shown in Figure 5, the decision hyperplane divides the graph into three parts, with each part containing a single class of samples. Obviously, the hyperplane generated by MCASVM in the output layer accomplishes the classification task and enhances the interpretability of the model. In comparison with DNNs that only produce output results, the hyperplanes represented by the output layer of MCASVM are more beneficial for decision-making. As previously mentioned, MCASVM and the traditional DNN can be realized by modifying the loss function model under the same structure. In the next section, the location of the decision hyperplane will be demonstrated after using the cross-entropy loss function to classify the dataset under the same hyperparameters and network structure settings.

**Figure 6:**
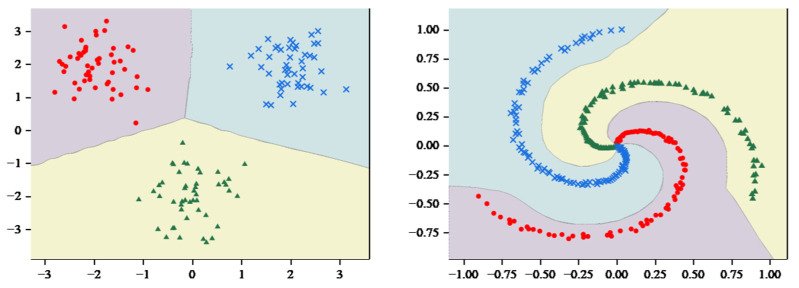
DNN Decision Region diagram.

As illustrated in Figure 6, the classification regions were compared by inputting the coordinates of the 2D image into the trained DNN model. The DNN also predicted the categories to which the different coordinate points belonged, which exhibited a high degree of similarity to the classification regions generated by MCASVM. However, a discrepancy was observed in the decision region for categorizing the spirals. It is conceivable that if the three spirals were extended, the decision space of MCASVM would accommodate a greater number of correctly categorized spiral sample points.

### 4.4. Probabilistically Calibrated Elimination Experiments

The effectiveness of the calibrated model can typically be visualized using reliability plots. In a reliability plot, the horizontal axis represents the intervals of the model’s prediction scores, dividing the model’s predictions into *M* intervals, with ${\;B}_{m}$ denoting the average level of prediction of the categorized model in the mth interval. For instance, the probability of [0, 0.1] can be assumed to be equally divided into 10 intervals. The initial interval corresponds to the set of samples with model output probability at [0, 0.1]. The mean prediction level of the model across the various intervals is depicted by Equation (13).

(13) $$conf\left(B_{m}\right)=\frac{1}{\left|B_{m}\right|}\sum_{i\in B_{m}}{\hat{p}}_{i}$$

The vertical axis of the reliability plot is indicative of the percentage of samples in these intervals that the model predicted correctly, as depicted in Equation (14).

(14) $$acc\left(B_{m}\right)=\frac{1}{\left|B_{m}\right|}\sum_{i\in B_{m}}1\left({\hat{y}}_{i}=y_{i}\right)$$

The indicator function, denoted by $𝟙\left(\cdot \right)$, takes on a value of 1 when the model’s prediction of *y* aligns with the true label, and a value of 0 otherwise. The discrepancy between the confidence level ascribed by the model and the true accuracy of the two is quantified by the expected calibration error (ECE), as depicted in Equation (15).

(15) $$ECE=\sum_{m=1}^{M}\frac{\left|B_{m}\right|}{n}\left|acc\left(B_{m}\right)-conf\left(B_{m}\right)\right|$$

**Figure 7:**
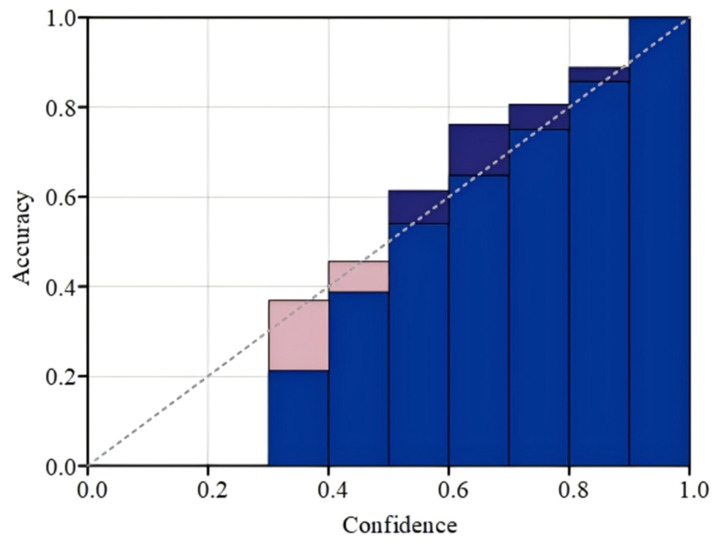
MCASVM uncalibrated outputs.

Reliability plots are generated before model calibration, subsequent to Platt scaling, and following temperature scaling calibration, respectively. The uncalibrated plot of the reliability of the model output is shown in Figure 7. The blue regions in these plots denote the true classification accuracy across the various intervals predicted by the model. The pink areas, along with the shaded regions that overlap with them, signify the discrepancy from perfect calibration.

**Figure 8:**
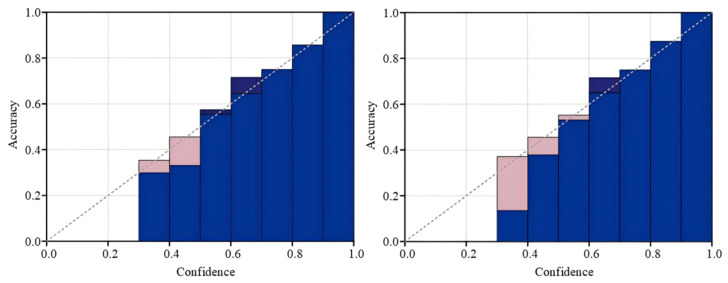
Reliability plot for Platt scaling and temperature calibration.

Following the calibration process, Platt scaling attains superior outcomes, thereby reducing the discrepancy between the optimal calibration and the ideal calibration. As shown in Figure 8 below, Platt scaling effectively adjusts the model’s output, ensuring that the output closely resembles the probability that the samples belong to a specific category.

## 5. Conclusions

With the development of artificial intelligence technology, AI painting tools have become an important auxiliary way for ordinary users to create art. However, it is still a challenge to systematically and objectively predict and identify the effect of the improvement of users’ art creation ability after using AI painting tools and its influencing factors. This paper explores the effects of AI painting tools on the artistic creation ability of ordinary users and the corresponding influencing factors by constructing MCASVM with a cross-entropy loss function. The MCASVM utilizes the structure of a neural network to implement the “one vs. rest” strategy of SVM, which gives full play to the advantages of deep learning models in feature extraction and complex pattern recognition while inheriting the characteristics of SVM that perform well on small sample data sets. In addition, the cross-loss function achieves the best performance by comparing different loss functions. There are many influencing factors that improve the creative ability of user groups, and the main ones include the basic characteristics of users, creative motivation, interest level, and social support. The experimental results show that AI painting tools have significantly improved the artistic creation ability of ordinary users, and the most core influencing factors are interest level and community support.

## Figures and Tables

**Table 1:** Characteristics and statistics of ACAAD.

	Characteristic	Classification	Training Set	Test Set	Total Sample
Basic	Gender	Male	4985	998	5983
		Female	5015	1002	6017
	Age	<18	2105	407	2512
		18~30	4993	1235	6228
		30~45	1367	186	1553
		45~60	1177	101	1278
		>60	358	71	429
	Degree	Specialist and below	2285	457	2742
		Bachelor	4632	1068	5700
		master	2375	415	2790
		doctor	708	60	768
Super	Artistic motivations	Strong	2765	552	3317
		Medium	4955	1093	6048
		Weak	2280	355	2635
	Interest level	Strong	1895	375	2270
		Medium	5567	1345	6912
		Weak	2538	280	2818
	Social support	Strong	2105	405	2510
		Medium	5789	1135	6924
		Weak	2106	460	2566

**Table 2:** Classification accuracy on ACAAD dataset.

	SVM	DNN		MCASVM(Ours)	
		One-Hidden-Layer	Two-Hidden-Layer	One-Hidden-Layer	Two-Hidden-Layer
ACAAD	0.9872 ± 0.0057	0.9849 ± 0.0038	0.9849 ± 0.0036	0.9924 ± 0.0011	0.9916 ± 0.0009

**Table 3:** Comparison between the cross-entropy loss function and L1 loss function.

MCASVM	L1		Cross-Entropy	
	One-Hidden-Layer	Two-Hidden-Layer	One-Hidden-Layer	Two-Hidden-Layer
ACAAD	0.9825 ± 0.0038	0.9821 ± 0.0036	0.9924 ± 0.0011	0.9916 ± 0.0009

**Table 4:** Comparison results of feature defaults.

Dataset	No Difference (%)	Reduce (%)	Rise (%)
Gender	91.7	3.6	4.7
Age	92.5	3.8	3.7
Degree	63.6	19.4	17
Artistic motivations	60.9	16.9	22.2
Interest level	45.1	7.7	**47.2**
Social support	42.6	16.1	**41.3**

Bold numbers represent a higher rate of increase in artistic creativity level after using AI drawing tools.

**Table 5:** Classification accuracy on BAID dataset.

SAAN	TANet	SVM	DNN		MCASVM(Ours)	
			One-Hidden-Layer	Two-Hidden-Layer	One-Hidden-Layer	Two-Hidden-Layer
0.7680	0.7545	0.7249	0.7216	0.7223	0.7596	0.7588

## Data Availability

All data generated or analyzed during this study are included in this article. The raw data are available from the corresponding author upon reasonable request.

## References

[1] Chen Z. (2024). Graph Adaptive Attention Network with Cross-Entropy. Entropy.

[2] Chen Z. (2024). (HTBNet) Arbitrary Shape Scene Text Detection with Binarization of Hyperbolic Tangent and Cross-Entropy. Entropy.

[3] Li J., Zhong J., Liu S., Fan X. (2024). Opportunities and Challenges in AI Painting: The Game between Artificial Intelligence and Humanity. J. Big Data Comput..

[4] Li J., Zhang B. (2022). The Application of Artificial Intelligence Technology in Art Teaching Taking Architectural Painting as an Example. Comput. Intell. Neurosci..

[5] Li L. The Impact of Artificial Intelligence Painting on Contemporary Art from Disco Diffusion’s Painting Creation Experiment. Proceedings of the 2022 International Conference on Frontiers of Artificial Intelligence and Machine Learning (FAIML).

[6] Liu X. (2020). Artistic Reflection on Artificial Intelligence Digital Painting. J. Phys. Conf. Ser..

[7] Rani S., Jining D., Shah D., Xaba S., Singh P.R. The Role of Artificial Intelligence in Art: A Comprehensive Review of a Generative Adversarial Network Portrait Painting. Proceedings of the International Conference on Intelligent Computing & Optimization.

[8] Wang J., Yuan X., Hu S., Lu Z. (2024). AI Paintings vs. Human Paintings? Deciphering Public Interactions and Perceptions towards AI-Generated Paintings on TikTok. arXiv.

[9] Xu J., Zhang X., Li H., Yoo C., Pan Y. (2023). Is Everyone an Artist? A Study on User Experience of AI-Based Painting System. Appl. Sci..

[10] Xu X. (2024). A Fuzzy Control Algorithm Based on Artificial Intelligence for the Fusion of Traditional Chinese Painting and AI Painting. Sci. Rep..

[11] Tian Y., Shi Y., Liu X. (2012). Recent advances on support vector machines research. Technol. Econ. Dev. Econ..

[12] Sebald D.J., Bucklew J.A. (2000). Support vector machine techniques for nonlinear equalization. IEEE Trans. Signal Process..

[13] Tang Y. (2013). Deep learning using support vector machines. CoRR.

[14] Zhang L., Zhou W., Jiao L. (2004). Wavelet support vector machine. IEEE Trans. Syst. Man Cybern. Part B (Cybern.).

[15] Bennett K.P., Blue J.A. (1998). A support vector machine approach to decision trees. Proceedings of the 1998 IEEE International Joint Conference on Neural Networks Proceedings. IEEE World Congress on Computational Intelligence (Cat. No. 98CH36227).

[16] Lin K.M., Lin C.J. (2003). A study on reduced support vector machines. IEEE Trans. Neural Netw..

[17] Mangasarian O.L., Musicant D.R. (1999). Successive overrelaxation for support vector machines. IEEE Trans. Neural Netw..

[18] Osuna E., Freund R., Girosi F. (1997). An improved training algorithm for support vector machines. Neural networks for signal processing VII. Proceedings of the 1997 IEEE Signal Processing Society Workshop.

[19] Suthaharan S., Suthaharan S. (2016). Support vector machine. Machine Learning Models and Algorithms for Big Data Classification: Thinking with Examples for Effective Learning.

[20] Hafiz A.M., Parah S.A., Bhat R.U.A. (2021). Attention mechanisms and deep learning for machine vision: A survey of the state of the art. arXiv.

[21] Niu Z., Zhong G., Yu H. (2021). A review on the attention mechanism of deep learning. Neurocomputing.

[22] Brauwers G., Frasincar F. (2021). A general survey on attention mechanisms in deep learning. IEEE Trans. Knowl. Data Eng..

[23] Rodriguez P., Velazquez D., Cucurull G., Gonfaus J.M., Roca F.X., Gonzalez J. (2019). Pay attention to the activations: A modular attention mechanism for fine-grained image recognition. IEEE Trans. Multimed..

[24] Liu Y., Shao Z., Hoffmann N. (2021). Global attention mechanism: Retain information to enhance channel-spatial interactions. arXiv.

[25] Cai W., Wei Z. (2020). Remote sensing image classification based on a cross-attention mechanism and graph convolution. IEEE Geosci. Remote Sens. Lett..

[26] Liu S., Lin T., He D., Li F., Wang M., Li X., Sun Z., Li Q., Ding E. Adaattn: Revisit attention mechanism in arbitrary neural style transfer. Proceedings of the IEEE/CVF International Conference on Computer Vision.

[27] Li Y., Zeng J., Shan S., Chen X. (2018). Occlusion aware facial expression recognition using CNN with attention mechanism. IEEE Trans. Image Process..

[28] Huang W., Zhou F. (2020). DA-CapsNet: Dual attention mechanism capsule network. Sci. Rep..

[29] Zhu X., Cheng D., Zhang Z., Lin S., Dai J. An empirical study of spatial attention mechanisms in deep networks. Proceedings of the IEEE/CVF International Conference on Computer Vision.

[30] DeRose J.F., Wang J., Berger M. (2020). Attention flows: Analyzing and comparing attention mechanisms in language models. IEEE Trans. Vis. Comput. Graph..

[31] Zhao Q., Liu J., Li Y., Zhang H. (2021). Semantic segmentation with attention mechanism for remote sensing images. IEEE Trans. Geosci. Remote Sens..

[32] Niu W., Sternberg R.J. (2001). Sternberg. Cultural influences on artistic creativity and its evaluation. Int. J. Psychol..

[33] Kaufman S.B., Quilty L.C., Grazioplene R.G., Hirsh J.B., Gray J.R., Peterson J.B., DeYoung C.G. (2016). Openness to experience and intellect differentially predict creative achievement in the arts and sciences. J. Personal..

[34] Joy S.P. (2005). Innovation motivation and artistic creativity. J. Creat. Behav..

[35] Asquith S.L., Wang X., Quintana D.S., Abraham A. (2024). Predictors of Change in Creative Thinking Abilities in Young People: A Longitudinal Study. J. Creat. Behav..

[36] Anderson R.C., Haney M., Pitts C., Porter L., Bousselot T. (2020). “Mistakes can be beautiful”: Creative engagement in arts integration for early adolescent learners. J. Creat. Behav..

[37] Mace M.A., Ward T. (2002). Modeling the creative process: A grounded theory analysis of creativity in the domain of art making. Creat. Res. J..

[38] Amabile T.M. (1979). Effects of external evaluation on artistic creativity. J. Personal. Soc. Psychol..

[39] Hu W., Wang X., Yi L.Y.X., Runco M.A. (2018). Creative self-efficacy as moderator of the influence of evaluation on artistic creativity. Int. J. Creat. Probl. Solving.

[40] Xue Y., Gu C., Wu J., Dai D.Y., Mu X., Zhou Z. (2020). The effects of extrinsic motivation on scientific and artistic creativity among middle school students. J. Creat. Behav..

[41] Rostan S.M. (1997). A study of young artists: The development of artistic talent and creativity. Creat. Res. J..

[42] Ershadi M., Winner E. (2020). Children’s creativity. Encycl. Creat..

[43] Jakkula V. (2006). Tutorial on Support Vector Machine (svm).

[44] Stanley R.P. (2007). An introduction to hyperplane arrangements. Geom. Comb..

[45] Bassano C., De Matteis G.M., Nardi P., Buratta M.M., Zeitani J., De Paulis R., Chiariello L. (2001). Mid-term follow-up of aortic root remodelling compared to Bentall operation. Eur. J. Cardio-Thorac. Surg..

[46] Mao A., Mohri M., Zhong Y. Cross-entropy loss functions: Theoretical analysis and applications. Proceedings of the International conference on Machine Learning, PMLR.

[47] Platt J. (1999). Probabilistic outputs for support vector machines and comparisons to regularized likelihood methods. Adv. Large Margin Classif..

[48] Guo C., Pleiss G., Sun Y., Weinberger K.Q. On calibration of modern neural networks. Proceedings of the International Conference on Machine Learning.

[49] Yi R., Tian H., Gu Z., Lai Y.K., Rosin P.L. Towards artistic image aesthetics assessment: A large-scale dataset and a new method. Proceedings of the IEEE/CVF Conference on Computer Vision and Pattern Recognition.

[50] Sze V., Chen Y.H., Yang T.J., Emer J.S. (2017). Efficient processing of deep neural networks: A tutorial and survey. Proc. IEEE.

[51] He S., Zhang Y., Xie R., Jiang D., Ming A. Rethinking Image Aesthetics Assessment: Models, Datasets and Benchmarks. Proceedings of the Thirty-First International Joint Conference on Artificial Intelligence.

[52] Pedregosa F., Varoquaux G., Gramfort A., Michel V., Thirion B., Grisel O., Blondel M., Prettenhofer P., Weiss R., Dubourg V. (2011). Scikit-learn: Machine learning in Python. J. Mach. Learn. Res..

